# A Regional DNA Barcode Library for Northern Rocky Mountain Arthropods to Support Biodiversity and Molecular Ecological Research

**DOI:** 10.1002/ece3.73150

**Published:** 2026-02-23

**Authors:** Mathew T. Seidensticker, Lorinda S. Bullington, Sergio E. Morales, Philip W. Ramsey

**Affiliations:** ^1^ Northern Rockies Research & Educational Services Florence Montana USA; ^2^ MPG Ranch Missoula Montana USA

**Keywords:** biodiversity, cytochrome c oxidase, DNA metabarcoding, insects, Rocky Mountain West

## Abstract

Arthropod DNA barcode reference libraries have advanced ecological research but remain incomplete in many areas, including the western United States. To improve coverage in the Northern Rocky Mountains, we developed the MPG Ranch Arthropod Library (MPG‐AL), a cytochrome oxidase I (COI) DNA barcode reference library for local arthropods in west‐central Montana. From 2017 to 2019, we collected 86,533 specimens from various habitats, generating 52,270 DNA barcodes for arthropod taxa from 38 orders, 389 families, 1668 genera, and 1793 species. A comparison of the MPG‐AL taxonomic coverage with a combined dataset of publicly accessible Montana arthropod DNA barcodes in the Barcode of Life Database (BOLD) and Montana Natural Heritage Program occurrence records revealed that the MPG‐AL added references representing 5154 Barcode Index Numbers (BINs) to BOLD. These additions mark a 280% increase for Montana arthropod DNA barcodes, including 1140 new BINs for taxa previously lacking reference barcode sequences in BOLD. The MPG‐AL provides a substantial foundation for establishing a comprehensive arthropod DNA barcode database in the Northern Rocky Mountain ecoregion. However, many taxa still lack reference barcode sequences, particularly in large, diverse insect orders. Future barcoding efforts are encouraged to expand regional taxonomic coverage through targeted sampling and collaborations with regional entomological collections. A comprehensive regional arthropod DNA barcode library will enhance our understanding of arthropod population trends and trophic relationships in the western United States amid persistent threats such as climate change, habitat loss, pesticides, and invasive species.

## Graphical abstract

We developed the MPG Ranch Arthropod Library (MPG‐AL), a COI DNA barcode reference library for arthropods of the Northern Rocky Mountains, to address gaps in barcode coverage for the western United States. From 86,533 specimens collected in Montana, we generated 52,270 DNA barcodes spanning 38 orders and 1793 species, adding 5154 Barcode Index Numbers (BINs) to BOLD—a 280% increase for Montana. The MPG‐AL establishes a strong foundation for regional biodiversity and molecular ecology research while highlighting the need for continued barcoding to fill remaining taxonomic gaps.

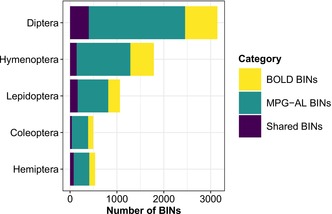

## 1 Introduction

There is widespread debate over whether the current biodiversity crisis signals an imminent or ongoing sixth mass extinction (Cowie et al. 2022; Wiens and Saban 2025). Arthropods account for most animal biodiversity, and reports of insect declines have raised significant concerns about the cascading impacts on ecosystems and biodiversity (Cardoso et al. 2020; Hallmann et al. 2017; Wagner et al. 2021). Surveying arthropod populations via traditional methods (i.e., collection and manual identification) is labor‐intensive and time‐consuming, hindering the timely development and implementation of strategies to mitigate negative biodiversity trends (Cristescu 2014). Accordingly, there is a need for cost‐efficiency, speed, and accuracy in establishing baselines for assessing and monitoring terrestrial arthropod biodiversity (deWaard et al. 2019). DNA metabarcoding, or the automated identification of multiple species simultaneously in environmental samples containing many organisms (e.g., bulk soil, water or fecal samples) (Taberlet et al. 2012) offers a cost‐effective, scalable, and robust method to address these concerns (Buchner et al. 2025).

DNA metabarcoding is widely used across disciplines to support biodiversity monitoring, ecological studies, diet analysis, and conservation (DeSalle and Goldstein 2019; Gostel and Kress 2022). The use of DNA metabarcoding in insect science has increased nearly 10‐fold, from less than 50 published studies per year in 2005 to nearly 250 in 2022 (Salis et al. 2024). Along with biodiversity assessments and species identification, DNA metabarcoding has also been used for identifying insect vectors of disease (Gariepy et al. 2012), economically important arthropods (Madden et al. 2016), plant–pollinator interactions (Newton et al. 2023; Thomsen and Sigsgaard 2019), and forensic entomology (Chimeno et al. 2019). However, the accuracy of these studies relies on the availability of high‐quality, well‐curated, and comprehensive DNA barcode reference libraries (Antil et al. 2023; Ekrem et al. 2007).

Numerous DNA barcode reference databases exist, some specialized for specific organisms like UNITE for fungi (Nilsson et al. 2019), MIDORI for metazoans (Machida et al. 2017), and COins for insects (Magoga et al. 2022), while others cover a broad range of taxa like NCBI GenBank (Sayers et al. 2019) and the Barcode of Life Data System (BOLD; Ratnasingham and Hebert 2007). BOLD is widely used for animal DNA metabarcoding studies because it maintains rigorous quality control by linking each DNA barcode to vouchered specimens and associated metadata (Baena‐Bejarano et al. 2023; Meiklejohn et al. 2019). BOLD also clusters sequences based on genetic similarity into operational taxonomic units (OTUs) called Barcode Index Numbers (BINs) that closely correspond to species or taxonomic groups (Ratnasingham and Hebert 2013), enabling standardized indexing of genetically similar taxa across studies.

The BOLD database includes over 13.5 million Arthropoda DNA barcodes in 793,918 BINs, representing 323,378 species, with insects comprising 90% (715,318) of the BINs and 88% (284,229) of the species. Yet these BINs account for only 27% of the estimated 1.2 million described terrestrial arthropods, 28% of described insects, and approximately 4.6% and 5% of the estimated 7 and 5.5 million terrestrial arthropod and insect species worldwide (Stork 2018). Coverage gaps are especially pronounced for large and diverse insect groups, such as Coleoptera, Diptera, and Hymenoptera (Csabai et al. 2023; Hendrich et al. 2015; Janko et al. 2024; Kjærandsen 2022; Lo et al. 2024).

Though Arthropoda DNA barcodes in BOLD are geographically widespread, more localized taxon coverage can enhance genus‐ and species‐level annotations. Prior studies (Fueyo et al. 2024) reported a nearly 33% increase in taxonomic assignments per sample for river macroinvertebrates from the Iberian Peninsula when sequences obtained from local taxa were added to the global BOLD database. Additionally, using a local database may reduce misidentifications (Ando et al. 2020), with some studies (Mugnai et al. 2023) showing improved taxonomic resolution by using a more targeted subset of barcodes. Misidentifications also occur as intraspecific similarity decreases with geographical distances, though this may be minimal (Huemer et al. 2018; Lukhtanov et al. 2009; Mutanen et al. 2012; Salis et al. 2024). Prior work (Mutanen et al. 2012) found that as the geographic scale of sampling increases, the overlap between intraspecific variation and interspecific divergence of barcodes becomes more likely. Taken together, this suggests that comparing query and reference sequences from smaller geographical scales yields higher classification precision.

Here, we present the MPG Ranch Arthropod Barcode Library (MPG‐AL), a COI DNA barcode reference library for local arthropods from a 15,000‐acre conservation property in west‐central Montana dedicated to restoring and sustaining biodiversity. We compared the library's taxonomic coverage to Montana arthropod barcode records in BOLD and the Montana Natural Heritage Program (MNHP). We discuss how the database contributes to research beyond our study area in the Northern Rocky Mountain ecoregion and encourage future efforts to expand arthropod DNA barcode coverage to support future biodiversity research.

## 2 Materials and Methods

### 2.1 Arthropod Collections

**FIGURE 1:**
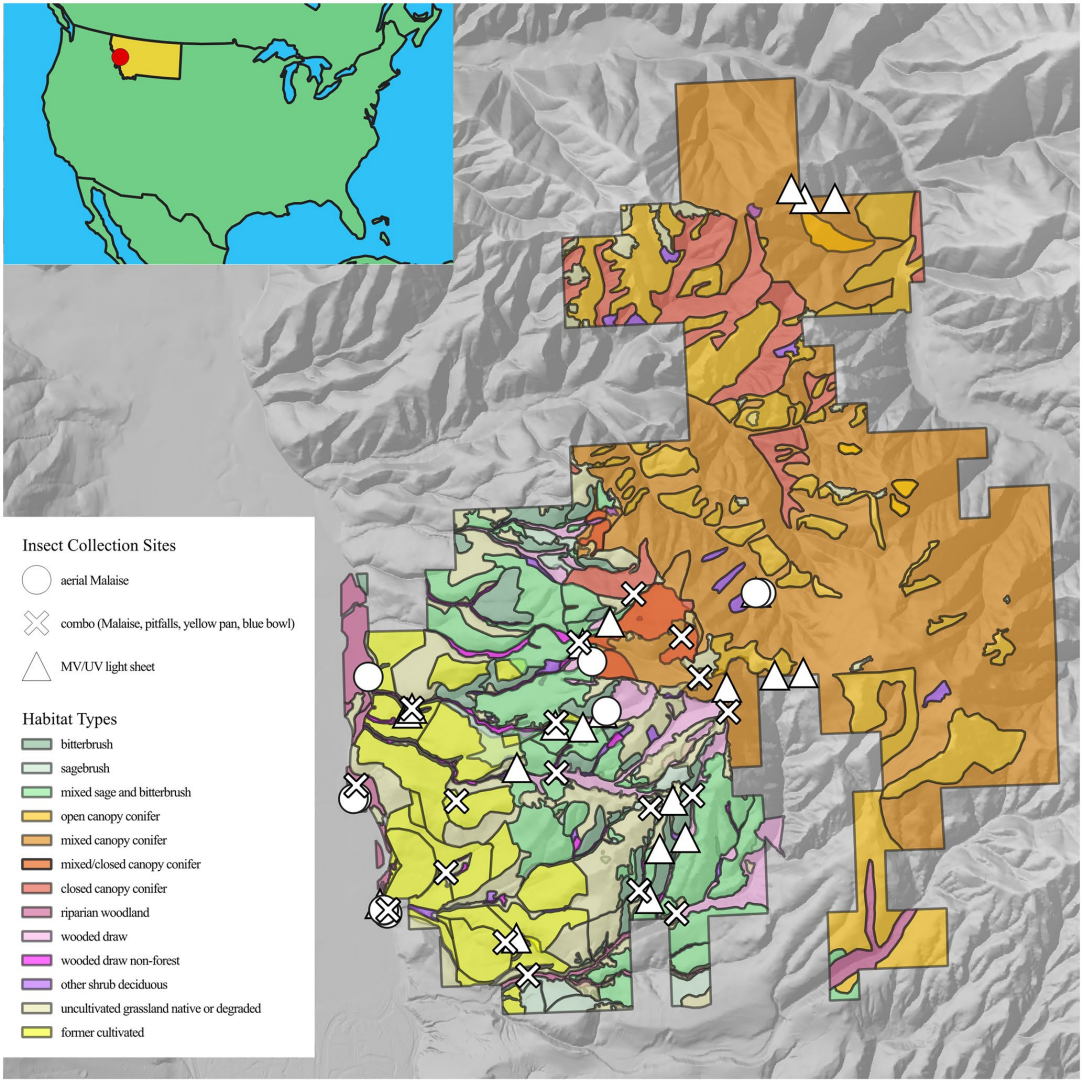
Locations of habitats sampled and methods used to collect arthropods for the MPG‐AL library.

All arthropod samplings occurred at MPG Ranch in Montana, USA, from 2017 to 2019 (Figure 1). MPG Ranch is a 15,000‐acre private conservation property in west‐central Montana with diverse habitats and elevations ranging from riparian floodplain to sage‐steppe to mid‐elevation mixed conifer forest. Arthropods were collected monthly at night from May through August of 2017–2018 using mercury vapor and black lights placed in front of a vertical white sheet and over 2‐ to 3‐day intervals of continuous passive collecting using manufactured standard‐size Sea, Land, and Air Malaise (SLAM) traps suspended from trees or poles. Sampling was expanded in 2019 to encompass a broader range of the insect community by clustering one flight‐intercept trap, three pitfall traps, and two pan traps in a 5 m radius at 18 sites across the study area, referred to as combination sites. Sites were not randomly selected. Sampling in 2017 and 2018 focused on sites where nocturnal aerial insectivores were captured and tracked, and their feces were collected for a molecular diet study (Bullington et al. 2021). The 2019 sites were chosen for accessibility along a private gravel road spanning an elevational gradient through the primary habitats on MPG Ranch. Sites were located between 50 and 400 m from the road.

The flight‐intercept traps (Russo et al. 2011) were modified to be approximately the same size as the SLAM traps (Supporting Information Appendix 1). Numerous large animals, including elk, bears, and approximately 50 feral horses, interact with human‐made objects on MPG Ranch. The feral horses, in particular, frequently damage equipment. Fabricated traps provided a composite system not available in commercially manufactured Malaise or SLAM traps, which allowed us to replace animal‐damaged parts. A 2‐in.‐deep, 16‐in.‐wide metal pan painted fluorescent yellow was placed beneath each flight‐intercept trap, and a blue bowl on a metal rod (8–10 in. above ground) plus three pitfall traps (0.5 m apart in a triangle) were set within 5 m of the trap. Pitfall traps were made by cutting the bottom 6 in. off clear 2‐L bottles, placing a clear Solo cup inside, then cutting the top 4 in. off the spout and inserting it upside down as a funnel. Traps were set in soil at or below ground level.

Combination sites were operated for 13 weeks from May through August 2019. Flight‐intercept traps were operated continuously over this period. Insects were caught in a 50/50 mix of propylene glycol and 95% EtOH inside the collection chamber. Catches were collected, and the preservation fluid was replenished every 7 days. Insects were caught in soapy water inside the pan and pitfall traps, and catches were retrieved after 48 h each week. Weekly catches from the three pitfall traps at each site were pooled into a single sample. Within several hours after retrieval, all catches were individually strained and washed with distilled water using a 25‐μm nylon filter, then placed into plastic (polyethylene) containers containing 95% ethanol and sealed with tight‐fitting screw‐on lids. Containers were labeled with collection dates, location data, and trap type before being stored in a freezer at −20°C for processing.

### 2.2 Specimen Processing and DNA Sequencing

All samples were sent to the Canadian Centre for DNA Barcoding (CCDB) for DNA extraction and sequencing (https://ccdb.ca/about‐us/). For specimens collected during 2017 and 2018, the authors first identified them to order morphologically based on experience and using the Key to the Orders of Hexapods (Borror et al. 1989), with the caveat that specimens keying to order Homoptera were classified as order Hemiptera reflecting current accepted phylogeny. This key follows the classic dichotomous structure of matching character descriptions provided in the key with specimen characters step‐by‐step until an order classification is reached. After classifying to order, we removed a 2–3 mm portion of a leg (femur) from large insects and put the samples in separate wells containing 30 μL 95% EtOH in 96‐well microplates provided by CCDB. If specimens were less than 2 mm, the entire specimen was placed in the well. The process was repeated until individual samples filled 95 wells, leaving the last well (96th) empty as a control. For each bulk sample collected from the flight‐intercept, pan, and pitfall traps in 2019, technicians at CCDB trained in insect identification first counted total abundance and weighed wet biomass before sorting samples morphologically by order and size in preparation for barcoding. Small specimens (< 2 mm) were placed directly into microplate wells whereas tissue samples were obtained from specimens larger than 2 mm. The authors nor CCDB technicians attempted to classify any specimens below order prior to barcoding.

DNA extraction, sequencing, and taxonomic assignments used established CCDB high‐throughput protocols (https://ccdb.ca/resources/). In summary, DNA was extracted using membrane‐based Glass Fiber protocols (Ivanova et al. 2006). DNA was assessed using a Bioanalyzer and Nanodrop system before PCR amplification of the cytochrome c oxidase subunit I (COI) gene using a single primer cocktail, C_LepFolF, C_LepFolR (Hernández‐Triana et al. 2014), using the following thermocycling protocol (initial denaturation for 2 min at 94°C, then 5 cycles of denaturation for 40 s at 94°C before annealing for 40 s at 45° and extension for 1 min at 72°C, then 35 cycles of denaturation for 40 s followed by annealing for 40 s at 51°C and extension for 1 min at 72°C, followed by final extension for 5 min at 72°C). Amplicons were then sequenced using SMRT (single molecule, real‐time) sequencing implemented on the SEQUEL platform (Hebert et al. 2018).

DNA sequences were first translated into amino acids and then compared against a Hidden Markov Model of the COI‐5P protein to identify gaps that provoke a frameshift or a stop codon and other sequencing or editing errors. Sequences with errors were manually re‐edited or re‐assembled from chromatogram trace files in CodonCode Aligner to correct errors made during initial sequence editing. Sequences with frameshifts were excluded. Resultant sequences were then uploaded to BOLD and assigned to Barcode Index Numbers (BINs) by the Refined Single Linkage (RESL) algorithm (Ratnasingham and Hebert 2013) if they met the following criteria: greater than 300 base pair (bp) coverage of the COI barcode region, less than 1% ambiguous bases, and no stop codon or contamination of the sequence. Sequences must include > 300 bp of the COI gene region for inclusion into an existing BIN and > 500 bp to establish a new BIN. DNA extracts produced during barcode analysis were stored in a DNA Archive, either in −80°C freezers or dried in trehalose or PVA‐based cryoprotectant and held in −20°C freezers. Associated vouchered specimens collected in 2019 were archived in a secure, microclimate‐controlled Specimen Archive (BIOUG) with specimen provenance data, timing of processing, and storage locator information digitized in BOLD's collection information management system (CIMS) (deWaard et al. 2019).

Taxonomic assignment and verification of BINs was completed following published workflows (deWaard et al. 2019). Generally, if a DNA barcode sequence was assigned to a BIN that contained specimens identified to a single family, genus, or species, it received that identification. If the record was assigned to a BIN with two or more members with taxonomic discordance, they were classified to the last shared taxonomic level. For example, if one BIN member was identified to genus A and the other to genus B, but both belonged to family C, the record would only be identified to the family level. In cases where the taxonomy of the BIN was only at the family level, the specimen sequences were entered into the BOLD Identification Engine (BOLD‐ID) and compared to the top 99 sequence matches from the complete COI DNA barcode library. Taxonomy was then applied using the same criteria as the BIN taxonomy assignment process. Species‐level identifications were assigned at ≥ 98% sequence similarity, genus‐level at ≥ 95% similarity, and family‐level at ≥ 90% similarity. When possible, morphological examination of voucher specimens was used to assign lower taxonomy of sequence‐based classifications higher than species. All failed sequencing samples, sequences with stop codons, records flagged as possible contamination or misidentifications, and sequences with less than 300 bp were removed from the final dataset. The full dataset can be accessed using the BOLD portal (https://doi.org/10.5883/DS‐MPGAL).

### 2.3 Estimating Taxonomic Coverage of the Reference Library

To gauge how well the MPG‐AL captured the taxonomic coverage of Arthropoda in Montana, we calculated the percentage of genera and species in each order included in the MPG‐AL by dividing the number of genera and species in the MPG‐AL by the number of genera and species in corresponding orders in a combined dataset of public arthropod DNA barcode records from Montana in the Barcode of Life Database (BOLD) and occurrence records from the Montana Natural Heritage Program (MNHP). Many of the records in the MPG‐AL were made public in a previous publication (Bullington et al. 2021). These records were removed before combining the remaining publicly available Montana arthropod records in BOLD with the MNHP occurrence records, hereafter referred to as the BOLD‐MNHP dataset (Table S1).

## 3 Results

**FIGURE 2:**
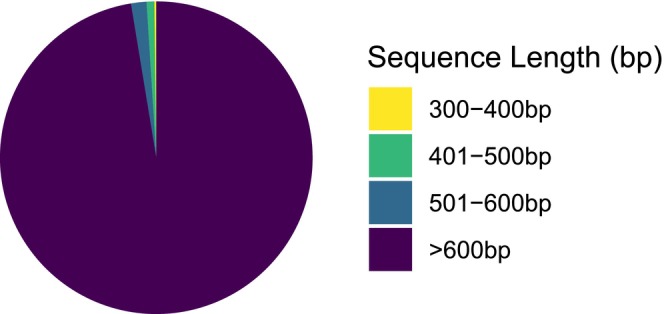
Composition of sequence base‐pair (bp) lengths in the MPG‐AL library.

Ninety‐nine percent of specimens were identified to the order level based on morphology before DNA extraction and sequencing. A total of 86,533 arthropod specimens were collected, and 64,757 were processed for DNA sequencing. The MPG‐AL includes 52,270 DNA barcodes from specimens collected at 50 sites in 10 habitats in spring and summer 2017–2019 (Table 1). Over 97% (50,903) of the sequences were > 600 bp, with 1% < 500 bp (Figure 2). As of November 1, 2024, 52,207 records were assigned to 6036 BINs, of which 1140 (19%) were unique to the MPG‐AL, creating new BINs for taxa previously lacking DNA barcodes in the BOLD database (Table S2). Sixty‐three records were not assigned to a BIN because the sequences did not match a known BIN and were less than the minimum 500 bp length required by the RESL algorithm to establish a new BIN on BOLD. At the time of writing, the taxonomy of 50,364 specimens (96%) was classified by BOLD representatives based solely on sequence data. Morphology was used to classify taxonomy lower than order for a small number of specimens (1073). Of these, 680 were Lepidoptera classified by the authors using retained voucher specimens, while the remaining 393 were classified by BOLD representatives. BOLD representatives used a combination of morphology and sequence data for 210 specimens and digital morphology and sequence data for 74 specimens to classify taxonomy lower than order. Digital morphology alone was used to lower taxonomy below order for 93 specimens. Four hundred and forty‐eight specimens had classifications below order with no identification method specified. Eight specimens remained classified at the original morphologically determined order level. There were no incongruences between subsequent sequence‐based classifications and the initial order level assignments.

**TABLE 1:** A summary of the sampling methods, total samples and species, and sampling frequency in each habitat sampled.

Habitat	Sampling frequency	Sampling methods	Seasons	No. of samples	No. of species
Bitterbrush	3	MV/UV light sheet	Spring Summer	35	26
Mixed sage/bitterbrush	21	MV/UV light sheet Combo	Spring Summer	7101	570
Open canopy conifer	1	MV/UV light sheet	Summer	30	21
Mixed canopy conifer	24	MV/UV light sheet Aerial Malaise Combo	Spring Summer	7857	606
Closed canopy conifer	2	Aerial Malaise	Summer	20	3
Riparian woodland	27	MV/UV light sheet Aerial Malaise Combo	Spring Summer	4199	445
Wooded draw	15	MV/UV light sheet Aerial Malaise Combo	Spring Summer	14,131	683
Wooded draw non‐forest	14	MV/UV light sheet Combo	Spring Summer	6855	429
Uncultivated grassland native or degraded	20	MV/UV light sheet Combo	Spring Summer	5707	525
Former cultivated	17	MV/UV light sheet Combo	Spring Summer	6335	355

Members of 18 new BINs had varying taxonomy. In all cases, members were classified in the same order, but some were identified only to order while others were identified to family or genus (Table S3). Since the MPG‐AL specimens were the only members of these BINs, we assigned the lower taxonomic classification for summary purposes. Twelve new BINs (1%) were classified to five spider species from Opiliones ( *Togwoteeus biceps* , 5 BINs) and Araneae ( *Habronattus hirsutus* , 3 BINs; *Paradosa ontariensis*, 2 BINs; *Hypsosinga rubens* , 2 BINs; and *Evarcha hoyi* , 2 BINs), likely representing polymorphism within these taxa. Thirty‐eight new BINs (3.3%) had identifications matching known species in a single BIN from Araneae (9 spp.), Diptera (7 spp.), Lepidoptera (5 spp.), Coleoptera (4 spp.), Hemiptera (4 spp.), Hymenoptera (4 spp.), and one species each from Dermaptera and Lithobiomorpha. The lowest classifications of the other 1090 unique BINs were 2.1% identified to class (23 BINs), 3.2% to order (35 BINs), 66.7% to family (728 BINs), 5.7% to subfamily (63 BINs), two BINs to tribe, and 239 BINs (21.9%) to genus. Diptera and Hymenoptera accounted for 682 (62%) of these new BINs, with 417 BINs distributed across 30 orders and three classes, accounting for ≤ 2% (Table S4). Specimens assigned to 212 existing BINs also had varying taxonomy (Table S5). Because these were existent BINs, some containing members of different taxa, we left these taxonomic assignments unchanged for summary purposes, resulting in the lowest classifications of the total 6036 BINs in the MPG‐AL as 52 (< 2%) identified to class, 20 (< 1%) to order, 1780 (30%) to family, 409 (7%) to subfamily, 1984 (31%) to genus, and 1793 (30%) to species.

**FIGURE 3:**
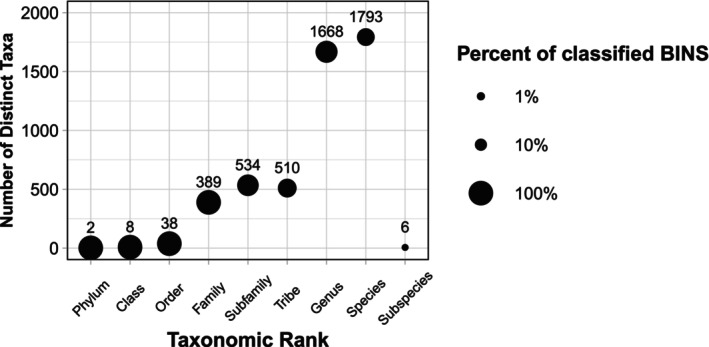
Taxonomic coverage and novelty of BINs based on the number of distinct taxa identified and percent classification to a known group at different taxonomic ranks. The size of the circles represents the percent classified, and the number in the circle represents the number of distinct taxa at each taxonomic rank.

The final MPG‐AL included taxa from 38 orders, 389 families, 1668 genera, and 1793 species (Figure 3 and Figure S1). Insects represented the bulk of the dataset in both number of specimens (48,674 [93%]) and BINs (5566 [92%]), with the remaining sequences assigned to Arachnida (2794 specimens, 370 BINs), Collembola (628 specimens, 75 BINs), Diplopoda (126 specimens, 12 BINs), Chilopoda (42 specimens, 10 BINs), Malacostraca (4 specimens, 1 BIN), Enoplea (2 specimens, 1 BIN), and Diplura (1 specimen, 1 BIN). Diptera were the most abundant insect group, representing 46% (23,956) of the specimens and 41% (2454) of the BINs. Hymenoptera were the second most abundant, representing 16% (8495) of specimens and 21% of (1286) BINs, followed by Hemiptera (11% of specimens, 6% of BINs), Lepidoptera (8% of specimens, 13% of BINs), and Coleoptera (5% of specimens, 6% of BINs). Orthoptera represented 2% of specimens but < 1% (37) of BINs. Combined, these six orders represented 95% and 97% of insect specimens and BINs, respectively, and 88% of total specimens and 89% of total BINs in the library.

The combined BOLD‐MNHP dataset covered 42 orders, 478 families, 3116 genera, and 7931 species, while the MPG‐AL contained 82 families, 503 genera, and 747 species not included in the BOLD‐MNHP dataset (Table 2). Four orders (Archaeognatha, Rhabura, Strepsiptera, and Nematoda) included in the MPG‐AL were absent from the BOLD‐MNHP dataset. Taxonomic coverage varied broadly by order (0%–100%) between the combined BOLD‐MNHP dataset and the MPG‐AL. The MPG‐AL included genus‐level coverage of ≥ 50% for 11 orders. However, overall genus‐level coverage was 37% and species‐level coverage was 13% (Table 3).

**TABLE 2:** Summary of families, genera, and species by order that are represented in the MPG‐AL library but absent in the BOLD‐MNHP dataset.

Order	# families not in BOLD‐MNHP dataset	# genera not in BOLD‐MNHP dataset	# species not in BOLD‐MNHP dataset
Diptera	11	176	278
Hymenoptera	8	140	127
Coleoptera	8	55	96
Hemiptera	4	46	76
Lepidoptera	5	28	84
Araneae	2	9	40
Trombidiformes	22	14	6
Thysanoptera	1	12	17
Psocodea	6	7	4
Mesostigmata	7	6	4
Neuroptera	1	4	5
Orthoptera	1	2	3
Entomobryomorpha	0	2	2
Symphypleona	1	1	1
Raphidioptera	0	0	2
Poduromorpha	2	0	0
Trichoptera	0	0	1
Julida	1	0	0
Chordeumatida	1	0	0
Isopoda	1	1	1
Total	82	503	747

**TABLE 3:** Estimated coverage of arthropod taxa in the MPG‐AL arthropod barcode reference library based on genera and species found in Montana as estimated from the Barcode of Life Database (BOLD) and Montana Natural Heritage Program (MNHP) databases (16,996 records).

Order	No. of genera in combined dataset	No. of genera in MPG‐AL shared with combined dataset (%)	No. of species in combined dataset	No. of species in MPG‐AL shared with combined dataset (%)	No. of BINs in MPG‐AL	No. of samples in MPG‐AL
Blattodea	2	0 (0)	2	0 (0)	0	0
Grylloblattodea	1	0 (0)	1	0 (0)	0	0
Megaloptera	1	0 (0)	2	0 (0)	0	0
Mecoptera	2	0 (0)	2	0 (0)	0	0
Pseudoscorpiones	3	0 (0)	3	0 (0)	1	1
Scorpiones	2	0 (0)	2	0 (0)	0	0
Solifugae	1	0 (0)	3	0 (0)	0	0
Scolopendromorpha	2	0 (0)	2	0 (0)	0	0
Geophilomorpha	1	0 (0)	1	0 (0)	0	0
Platydesmida	1	0 (0)	1	0 (0)	0	0
Julida	5	0 (0)	6	0 (0)	3	14
Chordeumatida	9	0 (0)	11	0 (0)	2	2
Isopoda	1	0 (0)	1	0 (0)	1	4
Sarcoptiformes	1	0 (0)	1	0 (0)	29	76
Siphonaptera	24	1 (4)	32	0 (0)	2	5
Ephemeroptera	60	5 (8)	159	5 (3)	12	57
Polydesmida	10	1 (10)	11	1 (9)	5	99
Odonata	35	3 (8)	108	1 (1)	3	20
Plecoptera	61	7 (11)	143	7 (5)	14	53
Lithobiomorpha	7	1 (14)	11	0 (0)	7	35
Coleoptera	603	127 (21)	1428	97 (7)	383	2530
Trichoptera	84	21 (25)	336	20 (6)	32	73
Ixodida	4	1 (25)	9	1 (11)	1	3
Orthoptera	78	21 (27)	159	16 (10)	37	1275
Entomobryomorpha	7	2 (29)	7	1 (14)	35	466
Poduromorpha	6	2 (33)	6	2 (33)	14	33
Mesostigmata	3	1 (33)	1	0 (0)	36	142
Trombidiformes	17	6 (35)	28	0 (0)	104	692
Opiliones	5	2 (40)	7	2 (29)	9	525
Hemiptera	318	128 (40)	600	69 (12)	410	5771
Lepidoptera	804	331 (41)	2764	474 (17)	813	4226
Araneae	161	72 (45)	435	91 (21)	170	1312
Dermaptera	2	1 (50)	2	1 (50)	4	364
Mantodea	2	1 (50)	3	1 (33)	1	2
Hymenoptera	278	148 (53)	841	123 (15)	1285	8495
Diptera	487	267 (55)	770	122 (16)	2454	23,956
Neuroptera	14	9 (64)	20	6 (30)	33	60
Thysanoptera	6	5 (83)	6	5 (83)	63	1347
Polyxendia	1	1 (100)	1	1 (100)	1	9
Raphidioptera	1	1 (100)	3	3 (100)	6	351
Symphyleona	2	2 (100)	1	0 (0)	26	129
Psocodea	2	2 (100)	2	0 (0)	14	24
Mermithida	—	—	—	—	1	2
Archaeognatha	—	—	—	—	2	54
Rhabdura	—	—	—	—	1	1
Strepsiptera	—	—	—	—	1	10
Total	3116	1168 (37)	7931	1050 (13)	6015	52,218

*Note:* Shading from lightest to darkest indicates taxonomic coverage at 0%, 1%–25%, 26%–49%, 50%–99%, and 100%. There were 49 records in 21 BINs and three records without a BIN identified to class only that are not included in this table.

**FIGURE 4:**
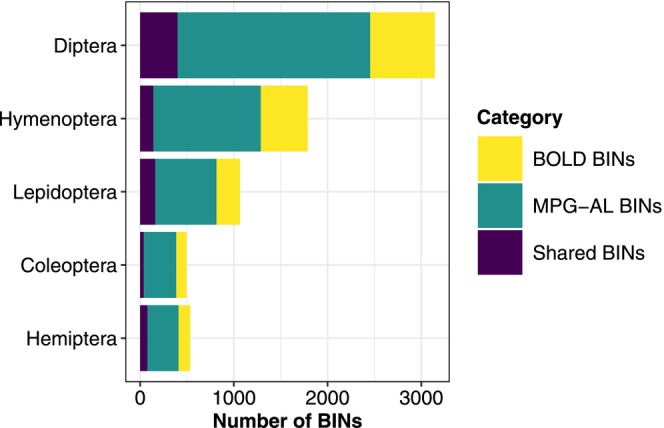
Distribution of Barcode Index Numbers (BINs) for records from Montana among five insect orders (Hemiptera, Coleoptera, Lepidoptera, Hymenoptera, and Diptera) in the MPG‐AL and BOLD databases. Each bar represents the number of BINs assigned to each order, with darker shades indicating BINs exclusive to the MPG‐AL database, medium shades indicating BINs in the BOLD but not MPG‐AL database, and lighter shades representing shared BINs present in both databases.

Despite the low overall taxonomic coverage of the MPG‐AL relative to the BOLD‐MNHP dataset, the MPG‐AL substantially increased the number of Montana arthropod DNA barcodes in BOLD. BOLD contains 2735 BINs for Montana arthropods, of which 882 (32%) are shared with the MPG‐AL, indicating that MPG‐AL added 5154 BINs, a nearly 280% increase. Eighty‐eight percent (4526) of these BINs were from five orders: Diptera, Hymenoptera, Lepidoptera, Coleoptera, and Hemiptera (Figure 4). The MPG‐AL also increased available arthropod DNA barcodes for the Northern Rocky Mountain ecoregion, which includes parts of Montana, Idaho, and Wyoming. BOLD currently contains 13,330 arthropod DNA barcodes clustered in 3803 BINs, currently representing 29 orders, 307 families, 11,421 genera, and 2248 species for this region. Twenty‐nine percent (1099) of these BINs are shared with the MPG‐AL, indicating that the MPG‐AL added 4939 BINs for the region. A list of the MNHP and BOLD records absent from the MPG‐AL is provided in Table S6.

## 4 Discussion

Information about insect diversity and population dynamics is sparse across the Northern Rocky Mountain ecoregion. The MPG‐AL substantially increased DNA barcode coverage in the region by 280%, providing data for 52,270 arthropods in 5154 BINs, including 1140 new BINs, which represent the first DNA barcodes for these taxa in BOLD. This effort significantly improves the potential of DNA metabarcoding for identification, monitoring, species discovery, and ecological study of local arthropod fauna in western Montana and the surrounding region. The MPG‐AL data have already been applied to new species descriptions and taxonomic revisions of beetles and flies (Figure S2) (Brunke et al. 2021; Savage and Sorokina 2021; Stuke and Levesque‐Beaudin 2023). These works included the first verified United States record of the adventive rove beetle *Oligota pumilio* (Brunke et al. 2021), the only United States paratype material examined for describing a new‐to‐science muscid fly *Drymeia hucketti* sp. nov. (Savage and Sorokina 2021), and holotype and paratype material examined for describing two new‐to‐science Nearctic carnid flies, *Meoneura pacifica* spec. nov. and *Meoneura tryionlanmisteri* spec. nov. (Stuke and Levesque‐Beaudin 2023). *M. pacifica* was described exclusively from voucher material and associated barcodes in the MPG‐AL. The 52 voucher specimens in the MPG‐AL matching 12 recognized but undescribed species from six genera of big‐headed flies (Pipunuculidae: *Cephalosphaera*, *Chalarus*, *Clistoabdominalis*, *Elmohardyia*, *Eudorylas*, *Tomosvaryella*) present further opportunities for integrative taxonomy. Members of two genera (*Chalarus*, *Eudorylas*) are known parasitoids of typical leafhoppers (Cicadellidae), while little is known about the life cycles of members in the other genera, offering research opportunities on host‐parasitoid interactions.

It is unknown whether members of the 1090 new BINs in the MPG‐AL identified at higher taxonomic levels match known species or represent new species until lower‐level classifications are determined, but the MPG‐AL voucher specimens and associated DNA barcodes in many of these BINs will support further taxonomic investigations. For example, the 197 new BINs of gall midges (Cecidomyiidae, Diptera) in the MPG‐AL may be particularly relevant for species discovery and taxonomic revision. Gall midges are an enormous group of plant‐feeding flies, with nearly 6000 named species. Since most adults are tiny, short‐lived, and often exhibit specific host‐plant affinities, gall midges may be the least‐known and largest fly family, with up to 90% of the taxa undescribed (Marshall 2012). Parasitic wasps are another large but poorly known group with many cryptic and undescribed species (Eagalle and Smith 2017). The MPG‐AL added voucher material and genetic data for 69 new BINs of these taxa, supporting species discovery, taxonomic revision, and host‐parasitoid research.

The MPG‐AL also provides valuable voucher material and genetic data for non‐insect taxa, as it contains DNA barcodes for many arachnids, springtails, and millipedes, which are often overlooked. For example, the MPG‐AL contains 13 new BINs in six oribatid mite families (order Oribatida). Many of these tiny arachnids are leaf litter feeders in the organic horizons of most soils and can reach densities of several hundred thousand individuals per square meter (Norton 1990). Despite being one of the most numerically dominant soil‐dwelling arthropod groups, and historically one of the better‐known soil‐dwelling acarine groups in Canada and Alaska, only 25% of the Canadian oribatid mite fauna is known at the species level (Behan‐Pelletier and Lindo 2019). Oribatids in certain families may also be endemic (Lindo 2018). The MPG‐AL material may enable species discovery, taxonomic revision, or ecological studies of oribatids' ubiquitous role in decomposition and nutrient cycling.

## 5 Limitations and Future Directions

Although the MPG‐AL added many Montana arthropod DNA barcodes to BOLD, substantial taxonomic gaps remain. This gap is evident from the low genus and species‐level coverage in the MPG‐AL compared to the BOLD‐MNHP dataset, particularly for diverse insect orders such as Diptera, Coleoptera, Hymenoptera, and Lepidoptera. However, BOLD uses internal and external taxonomic experts to curate records to the lowest possible classification, and we expect the taxonomic resolution of the MPG‐AL will improve. Still, the BOLD‐MNHP dataset used for comparison does not represent the true arthropod diversity in Montana, and the estimated taxonomic coverage in the MPG‐AL would be even lower than presented here if a more comprehensive list was available for comparison. The extent of the coverage gap would further increase if the true diversity of arthropods from Idaho and Wyoming were included for comparison. Gaps in the MPG‐AL may also bias downstream analysis for specific projects. For example, while the MPG‐AL supported over 75% of identifications in a molecular diet study of local nocturnal insectivores (Bullington et al. 2021), it may underperform in studies on beetles and diurnal pollinators, such as bumblebees and butterflies, which have limited taxonomic coverage in the MPG‐AL. These biases underscore the need for additional arthropod DNA barcode coverage in the Northern Rocky Mountain ecoregion.

A logical first step for improving regional arthropod DNA barcode coverage would be to integrate the 2704 arthropod BINs from Montana, Idaho, and Wyoming on BOLD with those in the MPG‐AL. Next, targeted sampling of missing taxa from the MNHP occurrence data is needed. This could be achieved through field collections or, perhaps more effectively, by DNA barcodes harvested from curated specimens in regional entomological collections. Natural history museums often house thousands of specimens representing numerous arthropod groups and can significantly enhance the availability of reliable entries for DNA barcode reference libraries (Mitchell 2015; Santos et al. 2023). Targeted sampling of missing taxa unavailable in museum collections, especially from diverse orders and important pollinator groups across different habitats and seasons, will also be required to generate a comprehensive DNA barcode reference library. Additional collecting should be done in collaboration with taxonomists for species identification and revision to increase the a priori taxonomic certainty of the DNA barcodes generated (Csabai et al. 2023; Macher et al. 2024).

The growing use of DNA metabarcoding in ecological research highlights its role in assessing insect biodiversity, monitoring populations, and understanding trophic interactions (Chua et al. 2023). The MPG‐AL represents a substantial increase in DNA barcode coverage of Northern Rocky Mountain arthropods. However, as our results suggest, significant work remains to achieve comprehensive arthropod barcode coverage in the Northern Rocky Mountain ecoregion. This limitation is challenging when assessing species‐specific trends, arthropod trophic interactions, and species discovery within and across the region's diverse ecosystems. Assimilating existing publicly available arthropod records from Montana, Idaho, and Wyoming on BOLD with the MPG‐AL will reduce the coverage gap, but targeted sampling in collaboration with taxonomists and regional insect museums is needed to attain complete arthropod DNA barcode coverage for the Northern Rocky Mountain ecoregion. Curated arthropod DNA barcode libraries enable repeatable studies of arthropod biodiversity, food webs, and pollinator interactions needed to inform conservation in the Northern Rocky Mountains and beyond.

## Author Contributions

**Mathew T. Seidensticker:** conceptualization (lead), data curation (equal), investigation (lead), writing – original draft (equal), writing – review and editing (equal). **Lorinda S. Bullington:** data curation (equal), writing – review and editing (equal). **Sergio E. Morales:** data curation (equal), formal analysis (equal), visualization (lead), writing – original draft (equal), writing – review and editing (equal). **Philip W. Ramsey:** conceptualization (supporting), funding acquisition (lead), project administration (supporting), writing – review and editing (equal).

## Funding

We thank MPG Ranch ownership for generously funding this research.

## Conflicts of Interest

The authors declare no conflicts of interest.

## Supporting information


**Appendix S1:** ece373150‐sup‐0001‐AppendixS1.xlsx.

**Appendix S2:** ece373150‐sup‐0002‐AppendixS2.docx.

## Data Availability

All barcode sequences and associated metadata are accessible through a public dataset, https://doi.org/10.5883/DS‐MPGAL. Specimens housed at BIOUG are available upon request to CCDB with author approval. Specimens collected in 2017 and 2018 were retained and vouchered by MTS in a personal collection.
