# Supplementary material for: A Regional DNA Barcode Library for Northern Rocky Mountain Arthropods to Support Biodiversity and Molecular Ecological Research
Source: Ecol Evol. 2026 Feb 23;16(2):e73150. doi: 10.1002/ece3.73150 (PMC12928087; doi:10.1002/ece3.73150)

**Supplemental Information for:**

**A Regional DNA Barcode Library for Northern Rocky Mountain Arthropods to Support Biodiversity and Molecular Ecological Research**

**Figure S1.** Taxonomic tree showing coverage across represented groups in the MPG Ranch Arthropod Library (MPG-AL).

**Figure S2** (a) first verified record of the adventive rove beetle, *Oligota pumilio,* in the United States (BIOUG50458-F11, by CBG Photography Group, licensed under CC BY 4.0 ([Deed - Attribution 4.0 International - Creative Commons](https://creativecommons.org/licenses/by/4.0/))); (b) one of 20 specimens exclusive to MPG-AL of *Meoneura pacifica* sp. nov (BIOUG51189-C12, CBG Photography Group, licensed under CC BY 4.0 ([Deed - Attribution 4.0 International - Creative Commons](https://creativecommons.org/licenses/by/4.0/))).


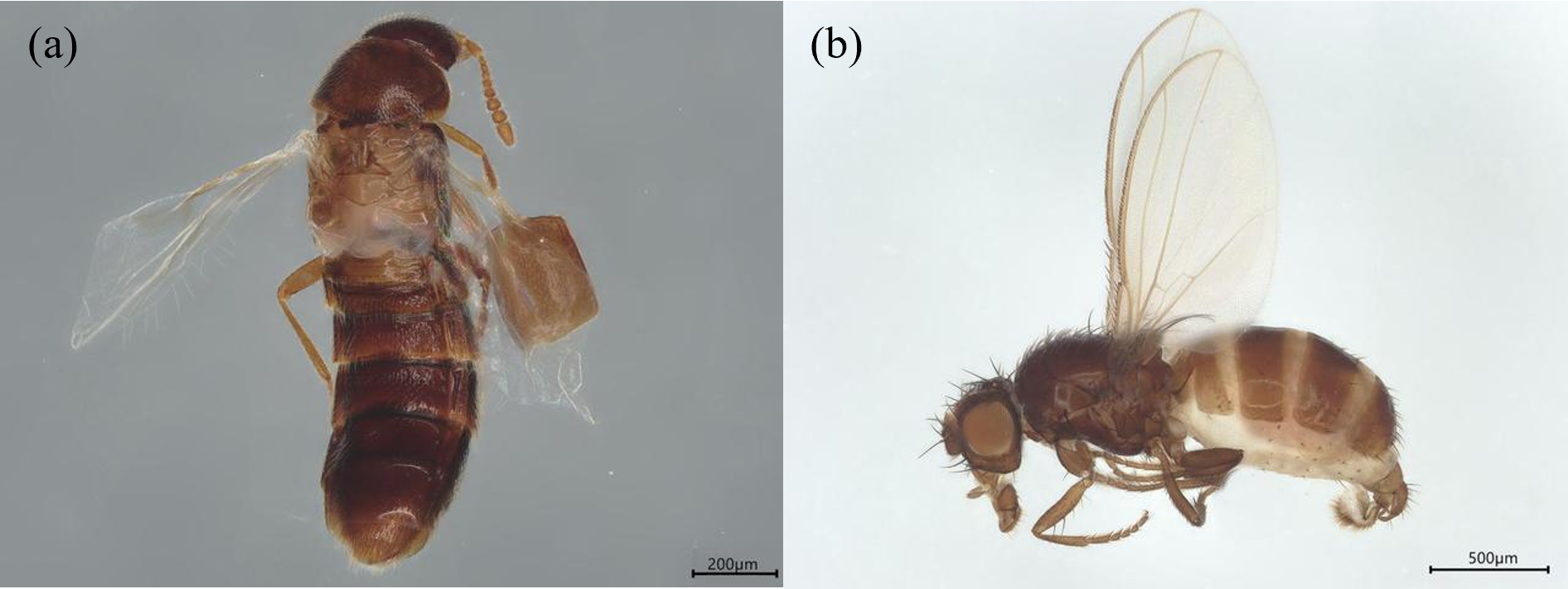


**Appendix 1.** Materials and instructions for assembling flight-intercept traps.

| Materials | Collection chamber | Flight-intercept |
| --- | --- | --- |
| (4 yds.) White polyester “no-see-um” netting |  | X |
| (3 yds.) Black polyester “no-see-um” netting |  | X |
| 18 oz. canvas (72-inches W by 48-inches L) |  | X |
| 24 oz. canvas (45-inches W by 48-inches L) |  | X |
| Velcro strips |  | X |
| (4) ½ inch rebar, 4-foot lengths |  | X |
| (4) ½ inch PVC pipe, 4-foot lengths |  | X |
| (4) 48-inch glass fiber plant support rods |  | X |
| (4) Velcro strips |  | X |
| UV thread |  | X |
| (8) 3/8-inch rubber O-rings |  | X |
| 1. 2-liter plastic bottle with lid | X |  |
| (1) Plastic (polyethylene) container with screw-on lid (~4.5 inches wide, ~3.75-inches deep) | X |  |
| Alcohol (EtOH) resistant sealant | X |  |

Assembly instructions:

Prep work and sewing time: 5.5 hours per trap.

1. Construction of flight-intercept bottom


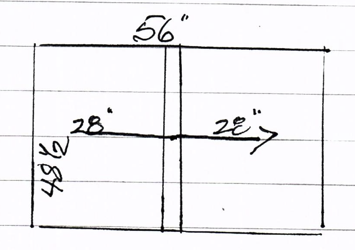


- 1. Cut two panels 56 inches wide by 48 inches height. Mark center at 28 inches.
  2. Put on center marking 1 inch binding centering on line pin.
  3. Bind top and bottom of the panels making sure the binding gets straight under the upper binding. Both panels with 1 inch binding need to match on the last step.
  4. Cut four casings (18 oz. canvas) 48 inches length by 5.5 inches width, mark, press, and bind.
  5. Put casings on raw edges of netting and sew centers together to make an “X”.

1. Construction of flight-intercept top


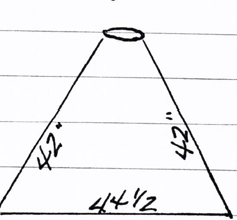


- 1. Cut and mark panels.
  2. Sew on triangle with both ends bound with 24 oz. canvas.
  3. Sew side seams together.
  4. Gently hold both cut sides to prevent gathering and stretching, about 3/8-inch seam.
  5. Sew down the 5-inch circle of tubing to the inside of the funnel shaped top.
  6. Double the netting and sew on pressed marked casing that has been bound at both ends on the bottom, following the stitching line.
  7. Turn bottom to top and sew casing together with double stitching.
  8. Sew top of casing closed on binding, stopping at marked end of panels.
  9. Do all four sides then bind the open ends of panels, then bind all around.
  10. Burn the raw edges of the binding before adding the Velcro hook and loop.

1. Construction of the collection chamber
   1. Cut the top of a two-liter (PET plastic) bottle approximately 4.5-inches down from the spout (where the curve begins to create a cone-like funnel).
   2. Remove the top of the screw-on lid so that only the threads remain to create a tube or opening that will connect the cone-like funnel to the collection chamber.
   3. Cut a hole in the bottom of the plastic collecting container that is approximately the same size (slightly larger) than the tube (lid) created in step B.
   4. Insert the tube (lid) into the hole at the bottom of the collecting chamber until the bottom of the lid and the bottom of the container are level. The top of the lid will protrude into the empty container.


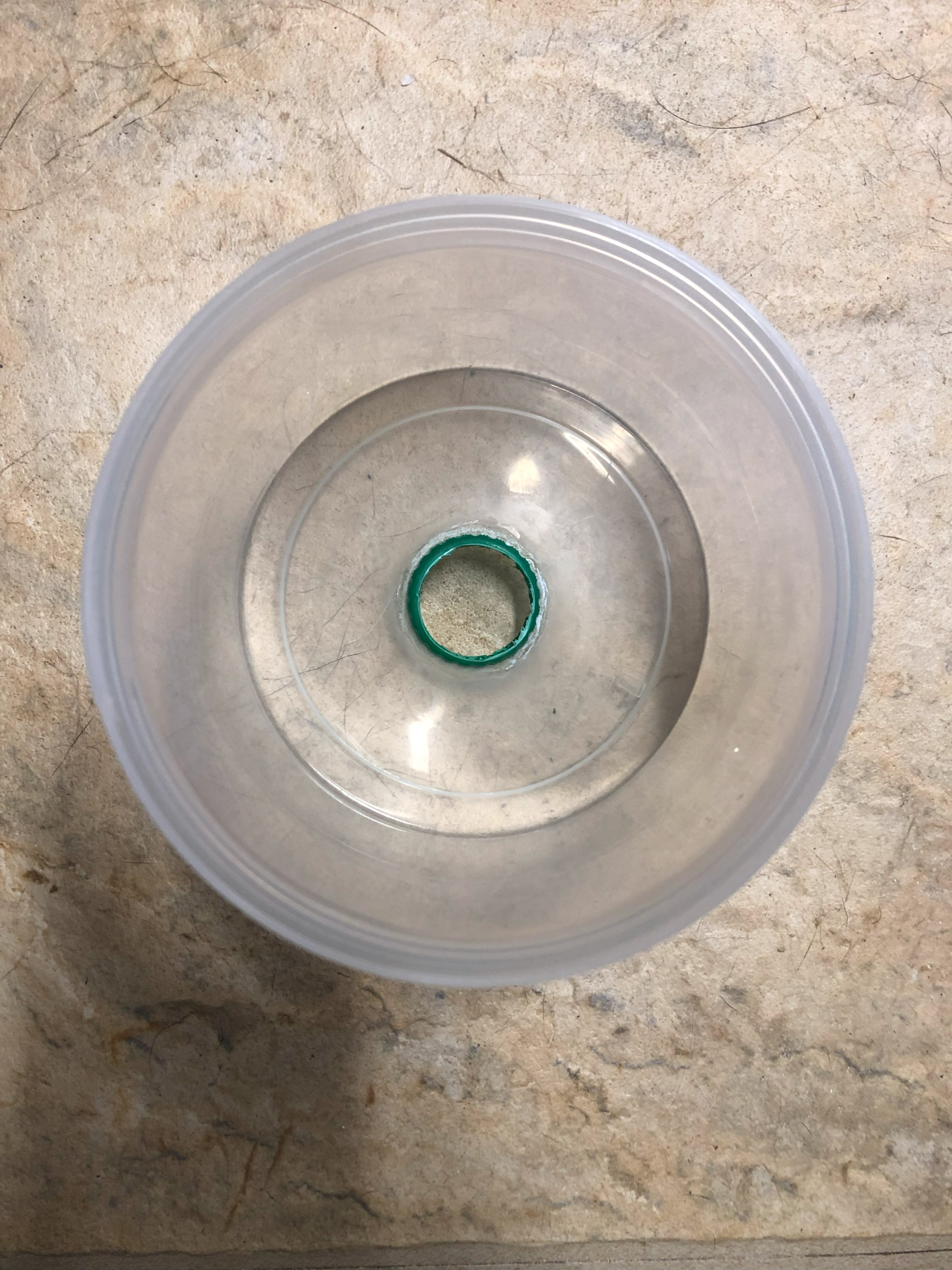


- 1. Use an alcohol resistant sealant to seal the edges around the lid on the bottom and inside the plastic container.
  2. Screw the cone-like funnel from the bottom of the container into the lid to create the collecting chamber.


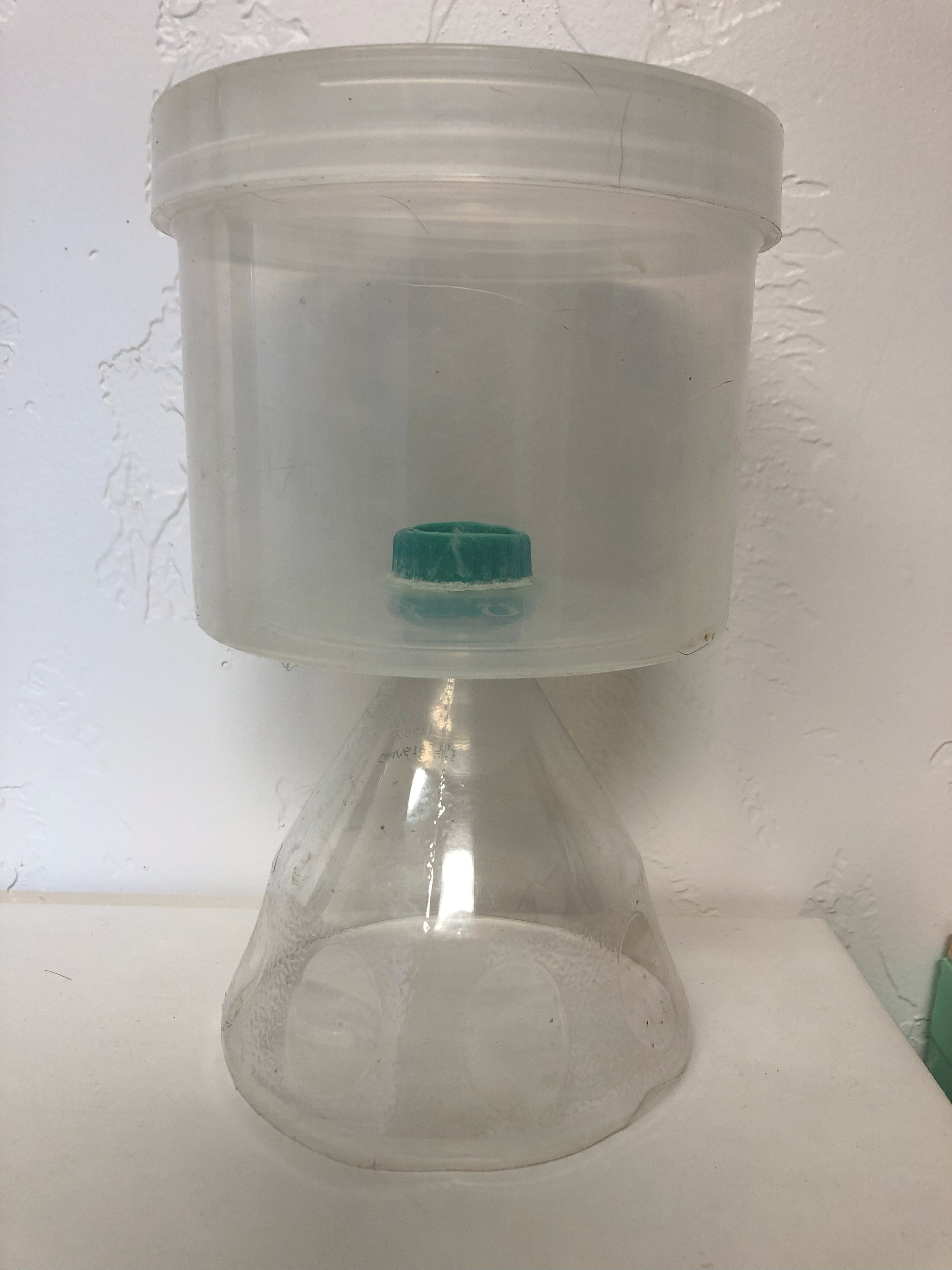


1. Assembling the trap in the field
   1. Insert the PVC tubes into the canvas casings of the trap bottom.
   2. Pound one rebar approximately 1 foot into the earth.
   3. Slide of the PVC tubes about half down the rebar.
   4. Stretch and align the opposite side of trap bottom with rebar, pound a second rebard into the earth and slide the PVC tube about halfway down that rebar.
   5. Repeat the process for the other ends of trap bottom.
   6. Slide the PVC tubes all the way down the rebar to ground level to fully secure the trap bottom.
   7. Slide casings down to expose 45° holes drilled through the top ends of each PVC tube.
   8. Insert garden stakes through the holes and into each of the four canvas casing on the trap top to make it stand up.
   9. Insert cone-like plastic funnel through the circle portion at the peak of the trap top so that the spout (thread) part is sticking up. From the top, thread the collecting container onto the funnel to complete setup.
   10. Guy lines can be secured around the top of each PVC tube and staked into the ground to add extra support.


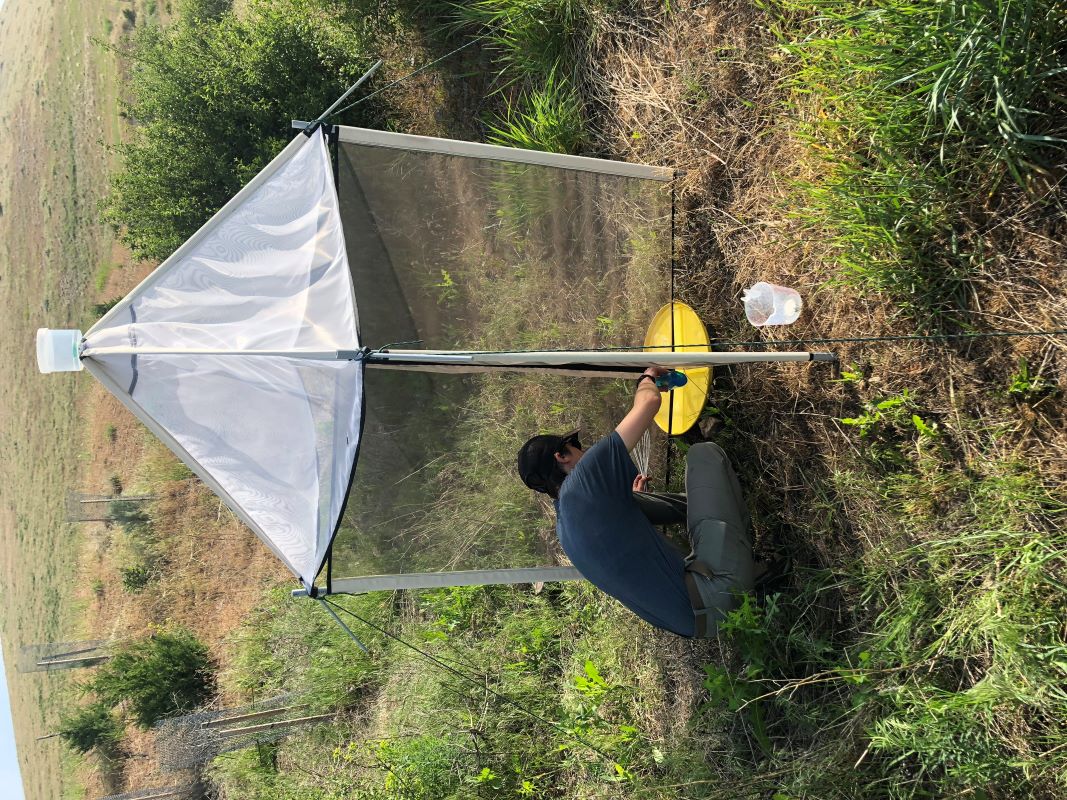

Supplement: Supplementary file 2 — Appendix S2: ece373150‐sup‐0002‐AppendixS2.docx. [file ECE3-16-e73150-s001.docx]
